# Identification of novel RNA secondary structures within the hepatitis C virus genome reveals a cooperative involvement in genome packaging

**DOI:** 10.1038/srep22952

**Published:** 2016-03-14

**Authors:** H. Stewart, R.J. Bingham, S. J. White, E. C. Dykeman, C. Zothner, A. K. Tuplin, P. G. Stockley, R. Twarock, M. Harris

**Affiliations:** 1 School of Molecular and Cellular Biology, Faculty of Biological Sciences, University of Leeds, Leeds, LS2 9JT, United Kingdom; 2 York Centre for Complex Systems Analysis, Departments of Mathematics and Biology, University of York, York, YO10 5DD, United Kingdom; 3 Astbury Centre for Structural Molecular Biology, Faculty of Biological Sciences, University of Leeds, Leeds, LS2 9JT, United Kingdom

## Abstract

The specific packaging of the hepatitis C virus (HCV) genome is hypothesised to be driven by Core-RNA interactions. To identify the regions of the viral genome involved in this process, we used SELEX (systematic evolution of ligands by exponential enrichment) to identify RNA aptamers which bind specifically to Core *in vitro*. Comparison of these aptamers to multiple HCV genomes revealed the presence of a conserved terminal loop motif within short RNA stem-loop structures. We postulated that interactions of these motifs, as well as sub-motifs which were present in HCV genomes at statistically significant levels, with the Core protein may drive virion assembly. We mutated 8 of these predicted motifs within the HCV infectious molecular clone JFH-1, thereby producing a range of mutant viruses predicted to possess altered RNA secondary structures. RNA replication and viral titre were unaltered in viruses possessing only one mutated structure. However, infectivity titres were decreased in viruses possessing a higher number of mutated regions. This work thus identified multiple novel RNA motifs which appear to contribute to genome packaging. We suggest that these structures act as cooperative packaging signals to drive specific RNA encapsidation during HCV assembly.

Hepatitis C virus (HCV) is the leading cause of chronic liver disease, hepatocellular carcinoma and liver transplants in the developed world. It is estimated that 170 million people are chronically infected worldwide, approximately 25% of whom will eventually present with liver cirrhosis. HCV therefore represents a significant health and economic burden. Direct-acting antivirals (DAAs) targeting 3 viral non-structural proteins are now available for treatment of HCV infection. The rates of viral clearance, however, differ significantly between genotypes and a low genetic barrier to resistance for certain DAAs means that viral escape is highly likely. Additionally, the pricing of the most recently available DAA (Sofosbuvir; a polymerase inhibitor) makes it unfeasible to treat the majority of patients in many countries. It is therefore apparent that further research into the basic virology of HCV is warranted.

HCV is of the family *Flaviviridae*, genus *Hepaciviridae* and possesses a ~9.6 kb single-stranded positive-sense RNA genome, encompassing highly structured untranslated flanking regions (UTRs). The 5′ UTR includes an internal ribosome entry site (IRES), which directs cap-independent translation of a single polyprotein that is subsequently cleaved into 10 constituent proteins: Core, E1, E2, p7, NS2, NS3, NS4A, NS4B, NS5A and NS5B. Although the non-structural (NS) proteins contribute to some degree to the assembly and release of the virion, Core, E1 and E2 play mostly structural roles^1^. The five C terminal NS proteins (NS3, NS4A, NS4B, NS5A and NS5B) were shown in 1999 to represent the minimal essential requirement for RNA replication of a subgenomic replicon^2^. These proteins and the ensuing RNA replication process have been available for targeted drug design much longer than the processes of virion assembly, RNA packaging and viral egress. The availability of a fully infectious viral molecular clone (JFH-1) means that these later stages can also be investigated in detail^3^. The role(s) of genomic RNA structures during genome encapsidation has not, however, been the subject of extensive research.

Genomes are packaged during formation of the nucleocapsid by the Core protein. Core is encoded by the 5′ region of the ORF and is released from the initially-translated polyprotein by host signal peptidase cleavage at the C terminus. Core is then further processed by signal peptide peptidase, resulting in a mature 21 kDa protein which forms homodimers and localises to cytosolic lipid droplets (for review see^1^). The NS proteins forming the RNA replication complex remain membrane-bound within an ER-derived membranous web where RNA replication occurs. There is accumulating evidence that the delivery of a nascent RNA by its cognate replication complex to the site of virion assembly is required for assembly initiation (for review see 4,5). However whether nucleocapsid formation and genome packaging occur at the lipid droplet, or whether this organelle merely acts as a storage site for Core prior to its relocalisation back to the cytoplasmic face of the ER (where assembly may occur), is a controversial topic^6^. Following assembly the nascent nucleocapsid buds into the ER, acquiring the lipid envelope and the associated E1-E2 viral glycoproteins prior to cellular egress (for review see 7).

It is apparent that the RNA replication and virion assembly processes are spatially distinct within the cellular microenvironment and interactions between the replication complex and the Core protein are required for efficient packaging^8^. Consequently, the packaging of replication-defective genomes (which are not presented by a replication complex) is notoriously inefficient in HCV. It is this inability to study assembly without concurrent replication which has hindered the identification of RNA structures contributing solely to assembly. The requirement for RNA presentation by the replication complex may prevent encapsidation of defective and/or partial HCV genome fragments by Core. The fact that cellular RNAs are excluded from this process indicates additional selective factors must contribute. For many positive-sense RNA viruses, the nucleocapsid assembly and genome packaging events occur simultaneously through recognition of a large stable RNA structure (the packaging signal, ψ) by their capsid protein. The essential role of these structural motifs within the viral RNA in directing genome encapsidation has long been recognised^9^. The fact that pre-formed “empty” HCV capsid-like particles have not been identified suggests that HCV utilises a similar mechanism of RNA recognition to initiate assembly^1^, although RNA-free capsid-like structures have been isolated from *in vitro* translation systems^10^. However, unlike prototypic packaging signals such as those found in the retroviruses, alphaviruses and coronaviruses, a single RNA structure which is both essential and sufficient to target non-viral RNAs to a nascent HCV nucleocapsid particle has not been identified.

It is possible that HCV utilises a recently discovered novel mechanism of genome encapsidation during nucleocapsid assembly, utilising multiple, relatively low-affinity structures termed Packaging Signals (PSs)^11^,^12^. We wished to investigate the possibility that similar specific secondary structures are present within HCV RNAs destined for packaging, and whether their interactions with Core cooperatively drive the RNA encapsidation and nucleocapsid assembly processes.

## Results

### Systematic evolution of ligands by exponential enrichment (SELEX) of RNA aptamers against Core

**Figure 1:**
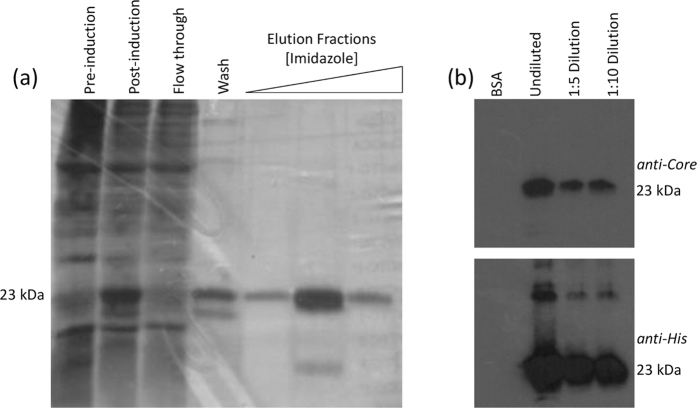
Expression and purification of Core D1. Core D1 was expressed in *E. coli* and purified via nickel affinity chromatography. The purity of the protein was assessed by silver staining SDS-PAGE of cell lysates and imidazole elution fractions (**a**) Immunoblots against the purified protein, compared to bovine serum albumin as a loading control, were performed using either an in-house polyclonal rabbit anti-Core serum (upper right panel) or a commercial anti-His murine monoclonal antibody (Invitrogen) (**b**).

Mature Core consists of two distinct domains: the N terminal domain 1 (D1, 124 residues), which is highly basic and binds RNA, and the hydrophobic C terminal domain 2 (D2, 50 residues) which possesses a membrane binding region. D2 stabilises Core on the surface of lipid droplets and exposes the hydrophilic D1 to the viral RNA, therefore it is essential for nucleocapsid formation in mammalian cells^13^. However, *in vitro* D1 is sufficient to form stable proteinase-resistant nucleocapsids when stabilised by interactions with nucleic acids^14^,^15^,^16^. We therefore prepared D1 as a SELEX target by recombinant protein expression. D1 HCV Core (JFH-1) was expressed in *E. coli* and purified by nickel affinity chromatography against an N-terminal hexahistidine tag. The identity of the his-tagged protein was confirmed by LC-MS/MS of Coomassie stained bands excised from an SDS-PAGE gel. The antigenicity of the final purified protein was also confirmed by immunoblotting for Core D1 and the hexahistidine tag (Fig. 1).

This protein was used for SELEX with a 2′OH RNA aptamer library encompassing a N35 random region. SELEX is an established process for the *in vitro* isolation of high-affinity aptamers: *in vitro* selected nucleic acid molecules which bind with high affinity and specificity to their target^17^,^18^. Each aptamer within a randomised library possesses a unique tertiary structure, depending on the series of stems, pseudoknots, kinks and/or bulges which are present in its most stable conformation. Each aptamer will bind with varying affinity to the ligand of interest. SELEX is therefore likely to enrich for aptamers with conserved conformation rather than a unique primary sequence.

### Locations and consensus motif of putative packaging signals (pPSs)

**Figure 2:**
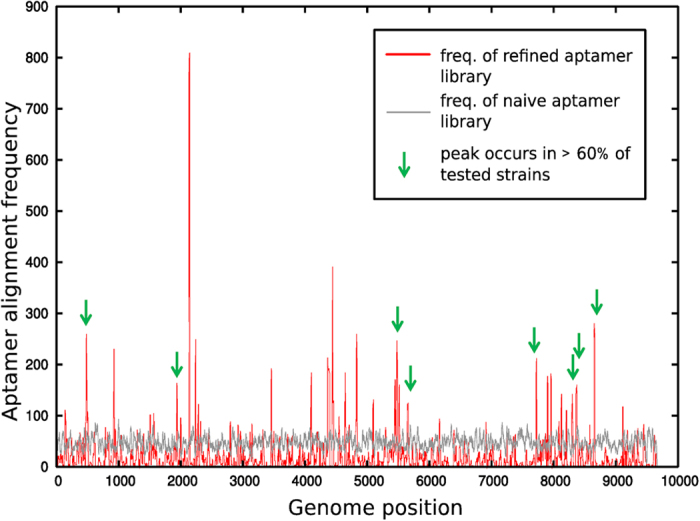
Packaging signals in HCV. Statistically significant alignments of the aptamer pool with the JFH-1 genome correspond to peaks in the red curve that are above the corresponding alignment of the naïve library (grey curve). Peaks occurring within 10 nucleotides of statistically significant peaks in at least 60% of the representative set of HCV genomes analysed here are marked by a green arrow.

The sequences present in the final round of SELEX were determined by Next Generation Sequencing (NGS) of the pool and the individual aptamer sequences were aligned to the JFH-1 genome (GenBank ID AB047639.1). It should be noted that known PSs in other ssRNA viruses are sequence/structure degenerate and their capsid/coat protein recognition sequences are discontinuous and minimal^12^,^19^,^20^. A consequence is that the pool of binders from SELEX should include a majority of oligonucleotides that, although matching essential features of HCV PSs, are unlikely to match extended regions of the genome. Despite this constraint, there are aptamers that match multiple sites within the genome and occur with statistical significance (a Bernoulli score of 12 or more; red peaks, Fig. 2) compared to the unselected naïve library (grey peaks, Fig. 2). This outcome suggests that the HCV genome does contain multiple PS sites. In order to test this hypothesis, the same procedure was applied to an additional 14 genotype 2 HCV genomes, i.e. they were compared to the aptamers selected against JFH-1, and a similar picture emerged. This suggests that these PS sites are evolutionarily conserved. Such peaks, within 10 nucleotides of the peaks in Fig. 2, which were conserved for at least 60% of these additional strains, are highlighted by green arrows (Fig. 2).

**Figure 3:**
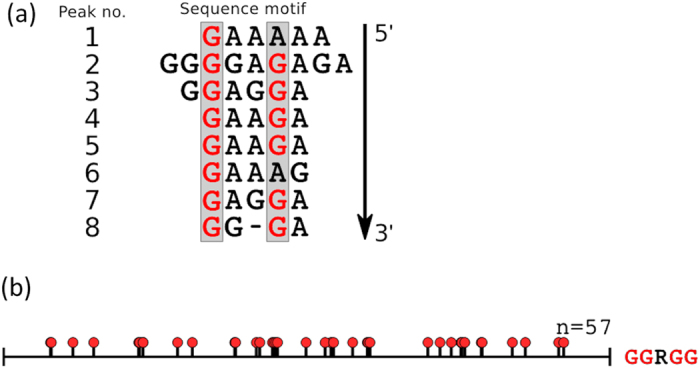
Identification and location of pPS recognition motifs. (**a**) Alignment of the loop portions of the stem-loops identified as a representative for each of the 8 conserved peaks in Fig. 2 (marked by a green arrow), revealing a dominant GRRGR motif, R denoting a purine. (**b**) Positions of stem-loops containing the GGRGG motif within the JFH-1 genome are shown as a suite of red lollipops.

For each of these conserved peaks the 30 nucleotides 5′ and 3′ to the peak nucleotide in the JFH-1 genome were extracted, and a range of possible secondary structure folds were considered via mFold^21^. A similarity analysis revealed the potential formation of stem-loops with similar loop motifs in each of these fragments. An alignment of the loop portions of these stem-loops is displayed in Fig. 3a. This analysis reveals a bias towards a GRRGR loop motif, R denoting a purine, suggesting that PSs should exhibit this motif or close variations thereof. In order to establish the statistical significance of this motif, we compared its occurrence in the loop portion of stem-loops in the wild-type genome with that in randomised versions of the genome, both for JFH-1 and for the other strain variants considered here. This confirmed a significant statistical bias in the wild-type genomes for this motif. We then interrogated sub-motifs of GRRGR in order to identify those with an even higher statistical significance, which revealed a GGRGG motif. The positions of stem-loops with this motif across the JFH-1 genome are displayed in Fig. 3b.

Modeling indicates that an ensemble of packaging signals with different degrees of affinity for capsid protein is required to promote efficient assembly^11^,^22^. Lower-affinity motifs may not necessarily be easily identifiable through a SELEX screen, yet their presence at statistically significant numbers within the wild-type genome would be indicative of a potential role in virus assembly. Based on previously-published models, the statistical bias observed for the GGRGG motif would be predicted to encompass at most 25% of the putative packaging signals (pPSs)^19^. Variations of the motif with statistical bias are the hallmark for PSs with intermediate or weaker affinity for the capsid protein. The same statistical analysis was therefore used to interrogate other variations of the GGRGR motif, in an effort to identify sub-motifs that were more frequent in the wild-type genome than in the randomised version. The motif GXRXR fulfilled these criteria (see Methods for details).

### Mutagenesis to disrupt pPS

**Figure 4:**
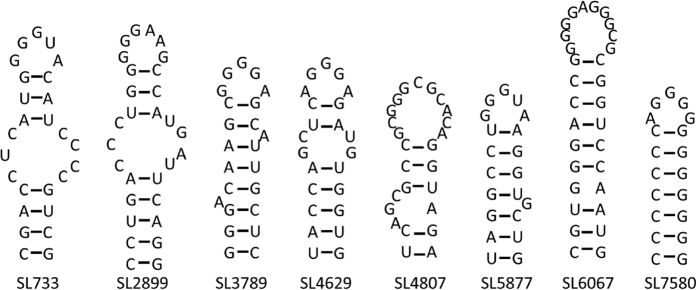
Predicted putative PS structures within the HCV genome. The local RNA structure of each of the regions of interest was modelled by mFold^21^. Motifs are named according to the first paired nucleotide within the JFH-1 genome, according to convention.

Since modeling suggests that pPSs act collectively to promote virus assembly, an ensemble of 8 pPSs was selected for mutagenesis. The region encoding NS5B was avoided as numerous studies have reported the existence of essential structures required for RNA replication; mutations in this region would render the virus replication-defective^23^,^24^. Modeling moreover implies that a combination of high and low affinity binders is important, i.e., a combination of pPSs following the GGRGG motif and those that exhibit other variations of the GRRGR motif: e.g. GXRXR^19^. Eight stem-loops were selected for mutagenesis, three of which contained the predicted highly conserved motif (GGRGG). The remaining five stem-loops contained the lower-affinity GXRXR motif identified above (Fig. 4). The stem-loops are numbered according to the first paired nucleotide within the context of the JFH-1 genome, according to standard nomenclature.

**Figure 5:**
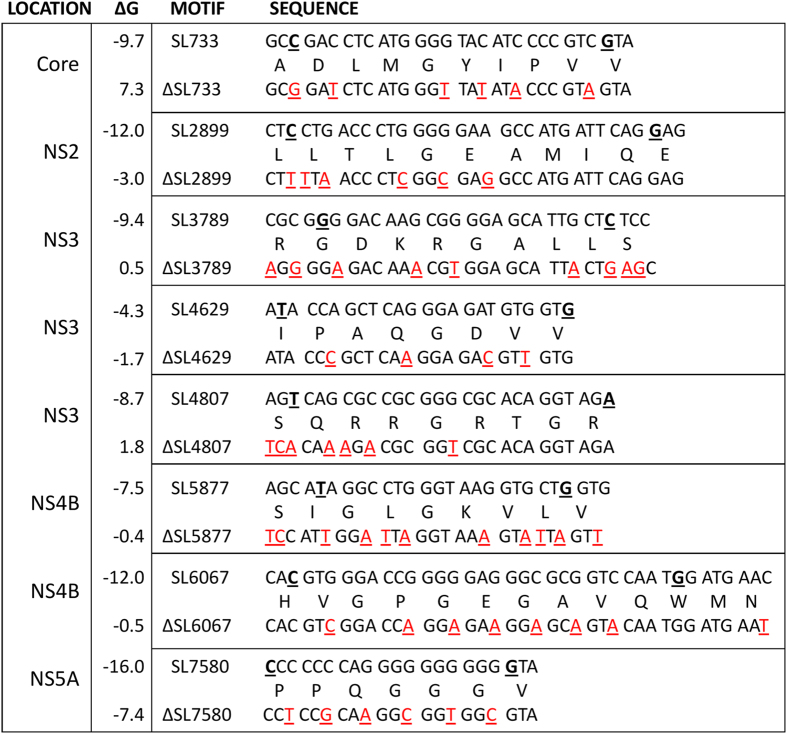
Silent mutations designed to disrupt the secondary structures of the pPSs within JFH-1. Multiple silent mutations (red, underlined) were incorporated into the pJFH-1 plasmid to disrupt the pPSs. The 5′ and 3′ nucleotides of the structures displayed from Fig. 4 are underlined in bold in the wildtype (upper) sequences; on occasion additional nucleotides immediately flanking these regions were mutated to maximise the disruption potential. Mutant genomes were named according to the disrupted stem-loop (ΔSL). The peptides encoded within each motif are described between the nucleotide sequences. ΔG refers to the free energy of the most stable predicted RNA secondary structure (kcal/mol) as calculated by mFold^21^.

A range of silent mutations were introduced into the ORF of HCV, using the infectious molecular clone pJFH-1. These were designed to disrupt the base pairing within the stems of the pPSs (Fig. 4). It was not possible in the majority of cases to completely ablate favourable folding energies for the formation of stem-loops and even mutant sites with unfavourable folding free energy might still form in the presence of a protein ligand such as Core. Eight mutant PSs were created, each possessing between 5 and 11 silent single nucleotide polymorphisms, collectively targeting an individual pPS (Fig. 5). These are referred to as the “single” pPS mutants, ΔSL733, ΔSL2899, ΔSL3789, ΔSL4629, ΔSL4807, ΔSL5877, ΔSL6067 and ΔSL7580. All 57 silent mutations were also combined into a single viral genome, termed Δ8XSL, in order to examine co-operative effects between the altered pPSs.

### Replication and translation are unaffected in pPS mutants

**Figure 6:**
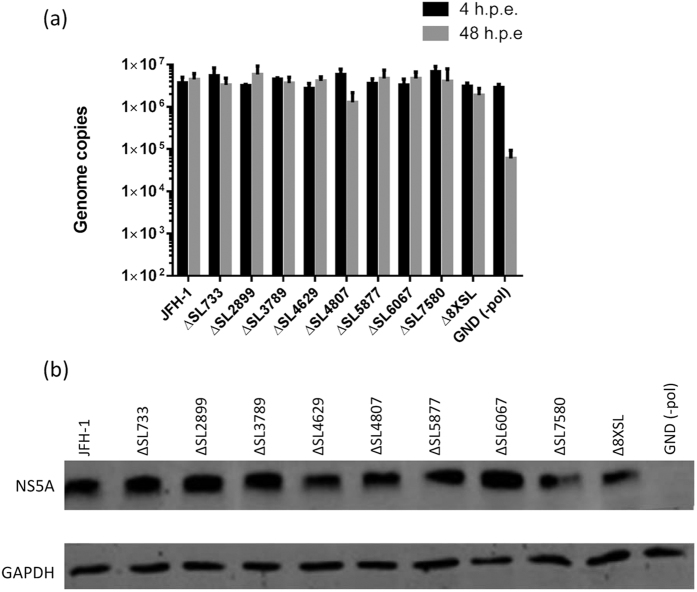
RNA replication and translation are unaffected in viral genomes containing disrupted pPSs. *In vitro* transcribed RNA was electroporated into naïve Huh-7 cells. At 4 and 48 h.p.e., RNA was extracted and quantified through qRT-PCR (**a**). Data represents the mean + SEM from three independent experiments. At 48 h.p.e., cells were lysed in Glasgow lysis buffer and 30 μg of each lysate was resolved by SDS-PAGE, followed by immunoblotting with NS5A-specific sheep polyclonal antiserum and a GAPDH commercial monoclonal antibody (**b**).

RNAs encoding all 8 single pPS mutant viruses, the Δ8XSL mutant, JFH-1 (wild-type) and JFH-1-GND (a polymerase-deficient control that is unable to undergo viral genome replication) were electroporated into Huh-7 cells. The intracellular RNA was quantified by qRT-PCR, at both 4 and 48 hours post electroporation (h.p.e) (Fig. 6a). Impaired replication was not observed in mutant viruses containing impaired pPSs, although ΔSL4807 possessed a slightly decreased replication rate which was not statistically significant. This was further confirmed by detection of the viral NS5A protein compared to cellular GAPDH expression via Western blot (Fig. 6b).

### Infectious viral titres are decreased in the Δ8XSL pPS mutant

**Figure 7:**
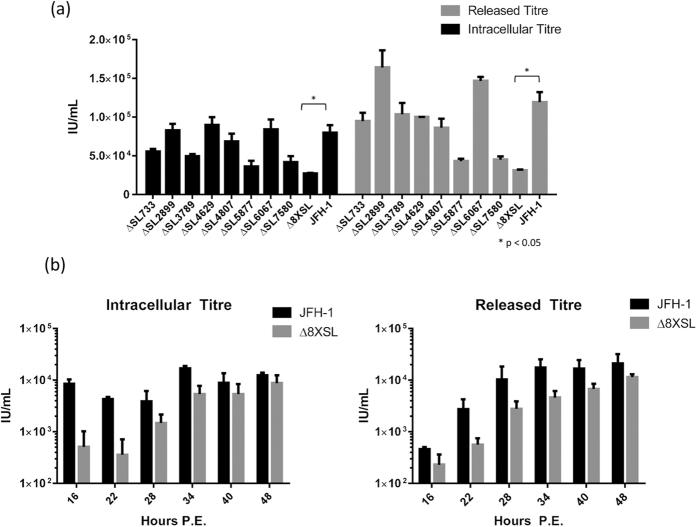
Viral titres are decreased in the multiple packaging signal mutant virus, Δ8XSL. 48 h.p.e., virus-containing supernatant and PBS lysates from electroporated cells were titrated upon naïve cells to determine the released and intracellular viral titres, respectively, of all single pPS mutant viruses, the multiple mutant virus Δ8XSL and wildtype JFH-1 (**a**). To assess the rate of virion formation and release, intracellular and released virus titres were also calculated for Δ8XSL and JFH-1 at multiple time points following electroporation (**b**). Data represents the mean + SEM from three independent experiments.

Both intracellular and released virus was titrated upon naïve Huh-7 cells and the infectious titre calculated in Infectious Units per millilitre (IU/mL) as previously described^25^. This reflects the number of infectious RNA-containing virions which had formed and/or been released from within the electroporated cells. Non-RNA containing particles, or aberrantly-assembled defective particles, therefore do not contribute to the viral titre as they are non-transmissible. At 48 h.p.e., only the Δ8XSL mutant virus displayed significantly decreased titres compared to the JFH-1 WT control, although ΔSL5877 and ΔSL7580 also appeared non-significantly decreased (Fig. 7a). The reduction in Δ8XSL infectious titre was consistent across the entire 48 hours, as evidenced by time course assays during which virus was harvested every 6 hours post-electroporation to assess the rate of virion formation and release (Fig. 7b). This provides evidence that although mutating each pPS independently has no apparent effect, the combined disruption of all 8 pPSs affects the late stages of the viral life cycle (assembly and/or egress) whilst not affecting RNA replication.

### RNA secondary structures are altered within the packaging signal mutants

To confirm that the introduced mutations caused structural alterations, *in vitro* transcripts from a section of the NS4B-coding region from JFH-1 (WT) and Δ8XSL mutant genomes were synthesised. RNA was folded and selective 2′-hydroxyl acylation analysed by primer extension (SHAPE) mapping was performed as previously described^24^. Briefly, folded RNA was treated with a compound which reacts preferentially with single-stranded, flexible regions. This reaction forms an irreversible adduct which causes premature termination during the subsequent reverse transcription of the RNA^26^. Primer extensions using radiolabelled primers were conducted and the resulting fragments were resolved on a 7% polyacrylamide denaturing gel. The terminations, indicative of a flexible nucleotide, were visualised and their reactivity normalised to that of a DMSO-treated control RNA. The specific location of such terminations is determined by comparison to ddNTP sequencing ladders.

**Figure 8:**
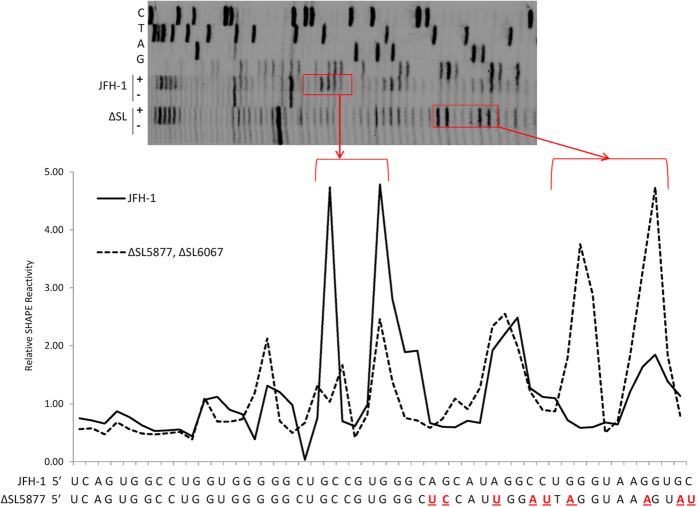
RNA secondary structure appears altered in subgenomic fragments containing disrupted pPSs. *In vitro* transcripts of the ~1.5 kb region encompassing SL5877 and SL6067 were synthesised from both JFH-1 and mutant templates. RNA was folded and subjected to structural analysis via SHAPE mapping. Following NMIA (+) or DMSO (−) treatment and reverse transcription, a primer designed to bind approximately 20 nucleotides downstream of SL5877 was used for primer extension. Products were resolved on a 7% denaturing polyacrylamide gel (upper panel) and the relative reactivity was quantified using SAFA^58^. Sequencing ladders were included to identify individual nucleotides, based upon the wildtype genome sequence.

A high SHAPE reactivity is indicative of a flexible nucleotide, available for acylation by the compound; hence only the terminal loop of a prototype stem-loop structure would appear as “reactive” nucleotides. The HCV genome is dynamic and multiple RNA conformations are potentially able to form, including kissing-loops, pseudoknots, G-quadruplexes and other long-range interactions^24^. Additionally the active folds of pPSs may only form in the presence of an RNA chaperone such as Core or NS5A, which is absent during SHAPE mapping. The SHAPE data therefore indicates whether the engineered mutations altered the RNA flexibility, rather than confirming the mFold-predicted structure of a particular motif. The reactivity profile across the region encompassing SL5877 provided evidence that the silent mutations had altered the flexibility of the mutant RNA compared to wildtype (Fig. 8).

Together our data indicates the 8 putative packaging signals which we identified may form within the HCV genome, presumably during assembly. A mutant genome unable to form such structures displays impaired viral infectivity whilst RNA replication is unaffected, indicating these motifs may interact specifically with Core. The synergistic effect of these individual interactions may ensure encapsidation of a single viral genome occurs during virion assembly, thereby preventing non-specific packaging of cellular RNAs.

## Discussion

The mechanism by which the HCV Core protein selectively binds and encapsidates viral genomic RNA during virion assembly is currently unknown. In contrast to other positive-strand RNA viruses^27^,^28^ there is no evidence for a single *cis*-acting RNA structure capable of directing packaging of both viral and non-viral RNAs to a nascent capsid particle. Therefore it is plausible that multiple RNA structures contribute to RNA-Core binding and act cooperatively to drive encapsidation and assembly. The presence of multiple, weak-affinity packaging signals in other RNA virus genomes has recently been reported^19^,^29^, which represents a novel mechanism for viral packaging in viruses which do not possess a readily identifiable prototypic packaging signal. Here, we provide evidence that the abrogation of short motifs (pPSs) located across the HCV genome has a significant effect on RNA encapsidation, manifested by a decrease in infectious titre. This is only apparent in the multiple pPS mutant (Δ8XSL), consistent with RNA packaging being a cooperative process in the *Hepaciviridae*. The fact that depletion of all 8 pPSs was required before a significant packaging phenotype was observed may explain why previous mutagenic screens have been unsuccessful in identifying these novel motifs^30^ – if the pPSs act synergistically, a threshold of ablation must be reached before significant phenotypic changes are observed. Additional low-affinity pPSs presumably also exist which were not mutated in this analysis. There are multiple occurrences of our described motifs within the JFH-1 genome which were not identified by our original aptamer screen; it may be that a large number of functionally redundant pPSs exist and hence are able to easily compensate for many mutations which may occur during the error-prone RNA replication process *in vivo*.

Previous models of assembly which utilise multiple contributing packaging signals predict that highly efficient capsid assembly occurs in the presence of one higher-affinity RNA structure; initial binding of this motif to the structural protein then instigates additional multimerisation and concurrent interactions with the lower-affinity packaging signals^22^. The relative affinities of each of the pPSs described in this study for the Core protein would therefore be of significant interest.

Prior to the development of the infectious JFH-1 cell culture system, models of HCV virion assembly relied upon either self-assembled capsid-like particles generated from purified recombinant protein^15^,^31^,^32^, or by expression of Core in yeast^31^, insect cells^33^ or bacterial systems^34^ followed by purification of the intracellular capsid-like particles. The formation of such regular capsid-like particles in these protocols requires the presence of structured RNA, proving that protein-protein interactions are insufficient to drive Core multimerisation. It is now recognised that these models do not reflect the assembly process in mammalian cells, therefore although investigating the binding kinetics of each pPS and its respective mutant would be of significant interest, future work may have to utilise alternatives to such *in vitro* assembly models. Additionally, the fact that intact HCV genomes cannot be packaged in *in vitro* assembly systems^15^ supports our model wherein the structures of interest may be formed temporarily and potentially only in certain scenarios, such as during presentation of nascent RNA within a replication complex. The NS5A protein (an essential component of the viral replication complex) possesses a broad RNA binding spectrum^35^ and interacts specifically with Core protein^36^; this combined with the fact that Core exhibits extensive RNA chaperone activity^37^,^38^ suggests a highly plausible model of RNA conformational alterations occurring during RNA transfer and subsequent assembly.

It is well established in the literature that Core binds to the viral 5′ UTR^15^,^39^,^40^,^41^,^42^, although this interaction is not sufficient to drive genome packaging^43^. We did not focus upon this region as its interactions with Core are already well-explored; the preferred Core-RNA binding motif is the IIId loop and this interaction inhibits IRES-mediated translation^40^,^44^. This motif is the only region of the IRES which possesses similar features to the stem-loops selected for mutagenesis; specifically, the internal bulge and a G-rich stretch within the unpaired terminal loop. This suggests that multiple structures, each containing this motif in their terminal loop, may be required to interact with Core during RNA encapsidation. As this IRES subdomain also forms long-range interactions with CREs in the NS5B coding region^45^,^46^, a model may be envisaged in which packaging, RNA replication and translation are mutually exclusive events, partially dictated by the ligand bound to the IRES IIId loop (Core, CRE RNA or the ribosomal complex).

The majority of our mutated pPSs are within the non-structural protein coding regions, with the exception of SL733. *Trans*-packaging of viral subgenomic replicons has been extensively documented (for review see)^47^ and it has been noted by other research groups that the functionality of these systems reflects the lack of essential *cis*-acting RNA elements within the Core to NS2 genomic region^48^. This is supported by our data wherein only 2 of the 8 mutated pPSs are located in this area (SL733 and SL2899); their removal may not reach a threshold level to affect packaging efficiency. *Trans*-packaging studies utilising baculovirus-mediated structural protein expression also found that although the presence of the NS2 protein improves the production of replicon-containing particles, this was most apparent when NS2 is expressed in *cis* (within the replication-cassette), rather than in *trans* (within the structural protein construct)^49^. Although this may be due to the protein-protein interactions required for virion assembly^50^, the fact that the pPS SL2899 is present upon this particular replicon may increase the efficiency of packaging, resulting in higher titres of virus-like particles in this system. In a parallel scenario, naturally occurring mutants with deletions spanning E1-p7 have been found in multiple patient samples; as these subgenomic mutants may be packaged and released^51^,^52^ an essential packaging signal is definitively not located in those coding regions.

Given the well-recognised roles of RNA structure in the HCV life cycle, it is important to highlight the novelty of the mutated structures in this report as many previously-annotated structures were not altered by our silent mutations, including the NS5B-located CREs^24^,^30^,^53^. In addition, the genome of HCV possesses genome-scale ordered RNA structure (GORS); a phenomenon amongst particular RNA viruses which exhibit extensive RNA structure across the entire genome^54^. Consequently the genome is constrained in its plasticity and evolutionary potential, in contrast to the predicted behaviour of a RNA virus. It has been suggested that GORS contributes to viral persistence or modulates host innate defence mechanisms. It therefore must be considered that these pPS structures may additionally contribute to the overall architecture of the HCV genome and may not be solely involved in the Core-RNA interactions during packaging – they may have multiple functions, requiring distinct structural conformations to be adopted at precise points during the viral replication cycle. The Δ8XSL mutant may represent a virus which has reached a threshold level of GORS interruption. However this is unlikely; an equivalent effect upon replication and assembly would be predicted if this were the case. It is therefore apparent that our analysis does not merely reflect RNA structures already annotated within the HCV genome, but rather the identification of novel structures utilised for Core-RNA binding.

## Materials and Methods

### Recombinant protein expression

Core (D1) of JFH-1 was cloned into the pET28B(+) construct (Novagen), in-frame with a N-terminal hexahistidine tag. Recombinant protein was expressed in BL21[DE3] cells (F– *omp*T *hsd*SB(rB– mB– *gal dcm* (DE3)). Luria broth media (1% glucose) was inoculated with a 1:1000 dilution of starter culture and maintained at 37 °C until an OD_600_ of 0.6–0.8 was reached. Protein expression was then induced by the addition of 100 mM isopropyl β-D-1-thiogalactopyranoside and cultures were maintained at 20 °C for 6 hours whilst shaking, before being harvested by centrifugation.

### Protein purification

Bacterial pellets were resuspended in lysis buffer (50 mM Tris-HCl pH 7.4, 200 mM NaCl, 1% Triton-X-100, 10% glycerol, 0.06% β-mercaptoethanol, 20 mM imidazole, protease inhibitors, DNase1, RNase A, pH 7.5) and lysed by sonication. The soluble fraction was filtered (0.45 μm) before being loaded on to an equilibrated Ni^2+^ Sepharose resin column (GE Healthcare). Resin was washed (50 mM Tris-HCl pH 7.4, 500 mM NaCl, 10% glycerol, 0.06% β-mercaptoethanol, 100 mM imidazole) and the protein was eluted into wash-based buffer with 500 mM imidazole. Protein was dialysed overnight into PBS and quantified by spectrometry. SDS-PAGE was conducted upon both bacterial lysates and eluted protein fractions according to previously described methods^55^ using 12% gels. Silver staining was conducted according to manufacturer’s instructions (SilverQuest kit, Invitrogen). Immunoblots were conducted with an in-house rabbit anti-core polyclonal sera (1:2000), commercial anti-His monoclonal antibody (1:1000, Invitrogen) and HRP-labelled secondary antibodies. For protein identification, Coomassie stained bands were excised, reduced, alkylated and digested with trypsin. The recovered peptides were analysed by LC-MS/MS and subsequent searching of the tandem MS data against the Uniprot sequence database.

### Systematic evolution of ligands by exponential enrichment (SELEX)

Recombinant D1 (1 mg) was immobilised via the His-tag to 12 mg of Dynabeads His-Tag Isolation and Pulldown magnetic beads (ThermoFisher Scientific) following the manufacturers protocol. Excess protein was removed with 3 washes of 100 mM sodium phosphate pH 8, 1 M NaCl, 0.02% (v/v) Tween-20. Bead immobilised protein was then washed 3 times with selection buffer (PBS, 0.05% (v/v) Tween-20, 1× Roche complete protease inhibitor per 50 mL). A Biomek 2000 laboratory automation work station (Beckman Coulter) was used to perform 10 rounds of *in vitro* SELEX as described previously^29^, using a N35 2′OH RNA library. This starting library was synthesised according to an experimentally and iteratively optimised protocol. It was purchased from AptaIT (Munich, Germany) who performed three rounds of synthesis, Next Generation Sequencing (NGS) and statistical analysis of NGS data from the library using the COMPAS software. In doing so the random region of the library has been optimised in respect of an equal distribution of nucleotide building blocks (25% of G, C, A & T, at each position), as well as in respect of a Gaussian distribution of motifs of length 4–8 nucleotides. An equal distribution of nucleotides increases the sequence space on the level of shorter motifs, which finally mediate binding to the target of interest. Consequently, the chance for a successful SELEX experiment increases with a homogeneous random region. Here the library was initially transcribed with a HiScribe T7 High Yield RNA transcription kit (New England Biolabs). Negative selections were carried out at each round of SELEX using bare His-Tag isolation and pulldown beads. The stringency of the aptamer-target interaction was raised by increasing the number of washes from 10 to 15 (slower off-rate) and by decreasing the amount of bead-immobilised protein by half (faster on-rate). Selections were carried out at 37 °C. Following the final round of SELEX, aptamers were analysed by NGS using a MiSeq Desktop Sequencer (Illumina). The SELEX library was prepared using the MiSeq reagent kit following the manufacturer’s protocol. Raw sequencing reads were processed using in-house scripts to identify sequences that contained correct 5′ and 3′ primers. Processed sequences were used for packaging signal identification (see below).

### Potential packaging signal identification

The following HCV genotype 2 genome sequences were extracted from GenBank: accession numbers AY232741; AY232730; AY746460; D00944; D50409; DQ364460; AB030907.1; AB031663.1; HM777359; AB047640; AF238481; AF238485; AF238486; AY232749; AB047640.1. The final selected aptamer library contained 112,117 unique sequences, each 35 nucleotides in length that were aligned against the JFH-1 genome as follows: each aptamer sequence was slid along the genome in increments of 1 nucleotide. For each such position of the reference frame, the subset of the aptamer sequence with the best alignment to the genome was identified according to the Bernoulli score B, which benchmarks the probability of a non-contiguous alignment to that of a contiguous alignment of B nucleotides. The Bernoulli scores for all reference frames of a given aptamer sequence in the library were rank-ordered, starting from the largest score, and all matches with the genome up to a Bernoulli score of 12 counted. The procedure was then repeated for the other aptamer sequences, and corresponding matches added, resulting in the peaks in Fig. 2.

### Identification of a consensus motif

This analysis was repeated for all genomes listed above. Peaks which occurred for at least 60% of the genomes within <10 nucleotides of each other were marked by a green arrow to indicate their conservation. These were used as a basis to identify the highly conserved pPS recognition motif. For this, sequences of 60 nucleotides, centred around the peak nucleotide, were extracted and all possible mFold predicted folds were considered. A similarity analysis of these stem-loops was performed, comparing both sequence and structure elements. The foldings of each peak area which had the highest degree of similarity with secondary structure elements in the other peaks was identified. This returned a stem-loop for each peak; an alignment of the corresponding terminal loop sequences is displayed in Fig. 2. In order to identify which features of the consensus motif are statistically significant, the number of occurrence of stem-loops with different types of sub-motifs in the genome was calculated and benchmarked against their occurrence in randomised versions of the genome. The consensus motif was GRRGR; this motif was 2.05 times more likely to occur in the wildtype than randomised genomes. In order to refine this motif, we performed similar statistical tests for submotifs of this consensus motif; we identified GGRGG and GXRXR motifs as being more likely to occur in the wildtype genome than in a randomised version, by a factor of 2.36 and 1.14, respectively, suggesting that pPSs with the former motif could correspond to high affinity PSs, and those with the latter motif could correspond to lower affinity PSs.

### Mutagenesis of pJFH-1

The DNA construct containing the JFH-1 viral genome (pJFH-1) and a replication-defective control mutant of this plasmid (termed pJFH-1-GND, possessing a GDD > GND mutation within the NS5B RNA-dependent-RNA-polymerase active site) have been described previously^3^. Mutagenesis of pJFH-1 to introduce silent mutations was performed by either site-directed mutagenesis (QuikChange, Agilent) or through overlap PCR utilising mutagenic primers (primer sequences available upon request). The entire genome of all mutant viruses was confirmed through Sanger sequencing to ensure additional mutations were not present.

### *In vitro* transcription

DNA constructs were linearised with *Xba*I, briefly treated with mungbean nuclease to degrade 3′ overhangs (New England Biolabs) and purified by phenol-chloroform extraction. Linearised DNA was used as a template for *in vitro* transcription to produce full-length HCV genomic RNA (RiboMAX Express; Promega, as per the manufacturer’s instructions). Following DNAse digestion, RNA transcripts were purified using silica-gel columns (RNEasy, Qiagen) and quantified by absorbance at 260 nm prior to transfection into mammalian cells.

### Mammalian cell culture

Huh-7 cells were maintained in Dulbecco’s modified Eagle’s medium (DMEM; Sigma) supplemented with 10% foetal bovine serum (FBS), 100 IU/mL penicillin, 100 μg/mL streptomycin, 25 mM HEPES and 1% (v/v) non-essential amino acids in a humidified incubator at 37 °C in 5% CO_2_^56^.

### Infectious HCV assays

6.0 × 10^6^ Huh-7 cells were electroporated with 5 μg of viral RNA at 975 μF and 260 V for 25 ms. Cells were resuspended in complete medium and seeded into multiple 6 well plates (6.0 × 10^5^ cells per well) (Corning). 4 hours post electroporation (h.p.e.), cells were harvested with TRIZol (Invitrogen Life Technologies) for RNA quantification. 24 h.p.e., all remaining wells were washed with PBS to remove excessive extracellular input RNA and the media replaced. 48 h.p.e., cells were harvested in TRIZol, protein lysis buffer or PBS, for RNA quantification, protein detection and intracellular virus titration, respectively. Virus supernatant was harvested at 48 h.p.e. for RNA quantification and released infectious virus titrations. Intracellular virus was isolated by 5 repeated freeze-thaw cycles of PBS cell lysates followed by clarification. Virus titres were calculated according to previously reported methods^25^. Briefly, either viral supernatant or cellular lysates were serially diluted two-fold upon naïve Huh-7 cells in 96 well plate format, seeded 6 hours prior to infection. At 48 hours post-infection, cells were washed, fixed with 4% paraformaldehyde and the viral NS5A protein was detected via indirect immunofluorescence. Automated counting was performed using the IncuCyte ZOOM (Essen Bioscience) using previously-described parameters^25^.

### Quantitative reverse-transcriptase PCR

RNA was purified by TRIZol extraction and diluted to 100 ng/uL. 200 ng was used in a Taqman probe-based one-step qRT-PCR according to previously-reported methods^57^. To quantify RNA, cycle threshold (Ct) values were converted to genome copies per 200ng of input RNA by comparison to a standard curve of serially-diluted pJFH-1 (10^2^ to 10^9^ copies).

### SDS-PAGE and Western blots

Following lysis in Glasgow lysis buffer (1% Triton-X-100, 120 mM KCl, 30 mM NaCl, 5 mM MgCl_2_, 10% glycerol, 10 mM PIPES-NaOH, pH 7.2 with protease inhibitors), lysates were clarified at 2000 × g for 2 minutes before protein concentration was determined by BCA assay (Pierce). 30 μg of cellular protein was separated by electrophoresis on 12% polyacrylamide gels and analysed by Western blot to detect NS5A and GAPDH as described previously^55^.

### RNA mapping by selective 2′ hydroxy acylation analysed by primer extension (SHAPE)

A 1527 nucleotide region across NS4B, flanked by the *Nsi*I and *SanD*I restriction enzyme sites, was cloned into pCRBlunt after PCR amplification from both pJFH-1 (WT) and Δ8XSL plasmid templates. The mutant insert therefore contained 19 silent point mutations ablating the predicted structures of SL5877 and SL6067. Subgenomic *in vitro* transcripts were then synthesised from a T7 promoter located upstream of the insert. RNA was heated to 95 °C for 3 minutes, before being refolded at 37 °C for 30 minutes in folding buffer (330 mM HEPES (pH 8.0), 20  mM MgCl_2_ and 330  mM NaCl). Folded RNA was modified with either 100 mM N-methylisatoic anhydride or DMSO at 37 °C for 45 minutes. Primer extension reactions were conducted as described previously^24^. Primers were labelled with γ-[33p]-ATP (PerkinElmer) and purified by ethanol precipitation prior to primer extension. cDNA extension products were separated by denaturing electrophoresis with a 7% polyacrylamide TBE gel (7 M urea), at 60 W for between 1.5 to 2.5 hours. Gels were visualised with a phosphoimager and the final images were processed to obtain normalised SHAPE reactivity using SAFA v1.1 software^58^.

## Additional Information

**How to cite this article**: Stewart, H. *et al*. Identification of novel RNA secondary structures within the hepatitis C virus genome reveals a cooperative involvement in genome packaging. *Sci. Rep*. **6**, 22952; doi: 10.1038/srep22952 (2016).
